# Renal inflammatory myofibroblastic tumor: a case report

**DOI:** 10.3389/fonc.2026.1819060

**Published:** 2026-04-20

**Authors:** Zhentao Zhang, Dawei Wang, Feihu Tang, Yulong Wang, Yuan Gao, Shunye Su

**Affiliations:** 1 School of Clinical Medicine, Shandong Second Medical University, Weifang, Shandong, China; 2 Department of Urinary Surgery, Weifang People’s Hospital, Weifang, Shandong, China

**Keywords:** ALK, immunohistochemistry, inflammatory myofibroblastic tumor, renal, surgery

## Abstract

**Objective:**

To investigate potential diagnostic approaches and therapeutic strategies for renal inflammatory myofibroblastic tumors (IMT).

**Methods:**

Renal IMT is a rare mesenchymal neoplasm with intermediate malignant potential arising within the urinary system. Its clinical presentation and imaging characteristics are nonspecific; thus, histopathological examination remains the diagnostic gold standard. This article presents a retrospective analysis of the clinical course of a single patient diagnosed with renal IMT.

**Patient:**

An elderly female presented with intermittent abdominal pain. Abdominal contrast-enhanced computed tomography (CT) revealed a solid mass located in the anterior lip of the right kidney. Following radical right nephrectomy, histopathological evaluation confirmed the diagnosis of renal IMT.

**Results:**

At the 3- and 6-month follow-up visits postoperatively, the patient showed no evidence of local recurrence or distant metastasis.

**Conclusion:**

Definitive diagnosis of renal IMT relies exclusively on histopathological assessments. For localized disease, complete surgical resection, preferably radical nephrectomy, is the treatment of choice. In advanced or unresectable cases, tyrosine kinase inhibitors and other targeted agents may be considered as potential therapeutic options.

## Introduction

Inflammatory myofibroblastic tumor (IMT) is a rare mesenchymal neoplasm characterized by intermediate malignant potential. It predominantly affects children, adolescents, and young adults, with no significant sex predilection (1). The lung is the most common anatomic site of involvement. Pulmonary IMT accounts for approximately one-third of all reported cases. Extrathoracic manifestations may occur in the abdomen, pelvis, retroperitoneum, internal organs and deep soft tissues of the head and neck (2).Within the urogenital system, the bladder is the most frequently involved site, whereas renal involvement is exceedingly rare (3). To date, only 49 cases of renal IMT between 1972 and 2019 have been documented in the literature.

## Study case

**Figure 1:**
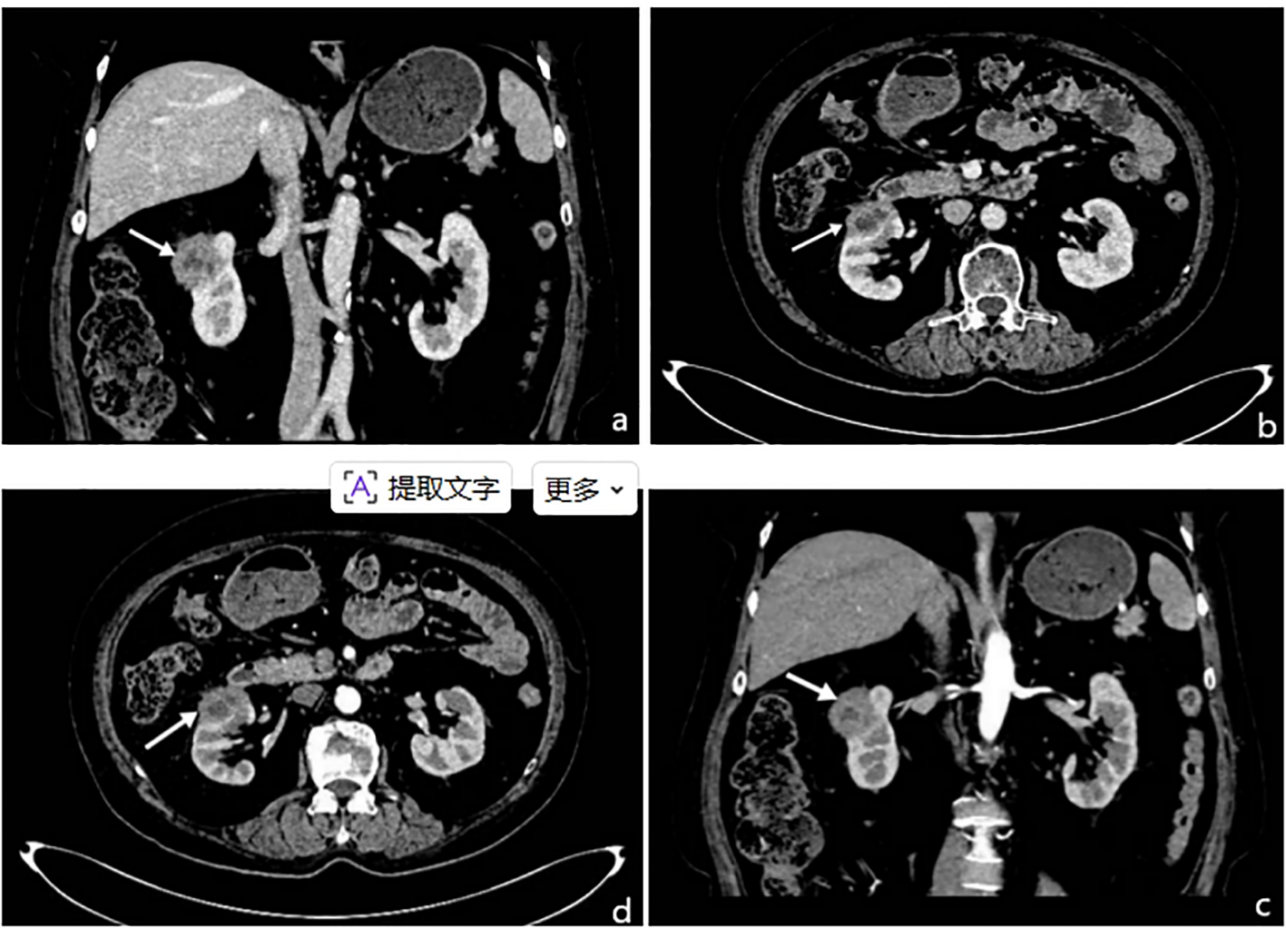
Image 1 displays contrast-enhanced computed tomography (CT) images of IMT involving the kidneys acquired during the venous phase **(A, C)** and arterial phase **(C, D)** following intravenous administration of iodinated contrast medium. Arrows in the Image indicate the tumor locations. **(A)** presents a coronal reconstruction of the renal IMT in the venous phase and **(B)** shows the corresponding axial (horizontal) image in the same phase. **(C)** depicts the axial image of the IMT in the arterial phase, while **(D)** illustrates the coronal reconstruction in the arterial phase. In **(A, B)** both kidneys exhibit normal morphology and size. A well-defined nodular lesion measuring approximately 3.5 cm × 3.1 cm is identified at the upper pole of the right kidney. The lesion showed demonstrates heterogeneous attenuation and ill-defined margins. It exhibits progressive and sustained enhancement throughout the venous and delayed phases. In images c and d, the lesion margin shows low enhancement in the arterial phase of IMT and the central low-density area shows no obvious enhancement.

**Figure 2:**
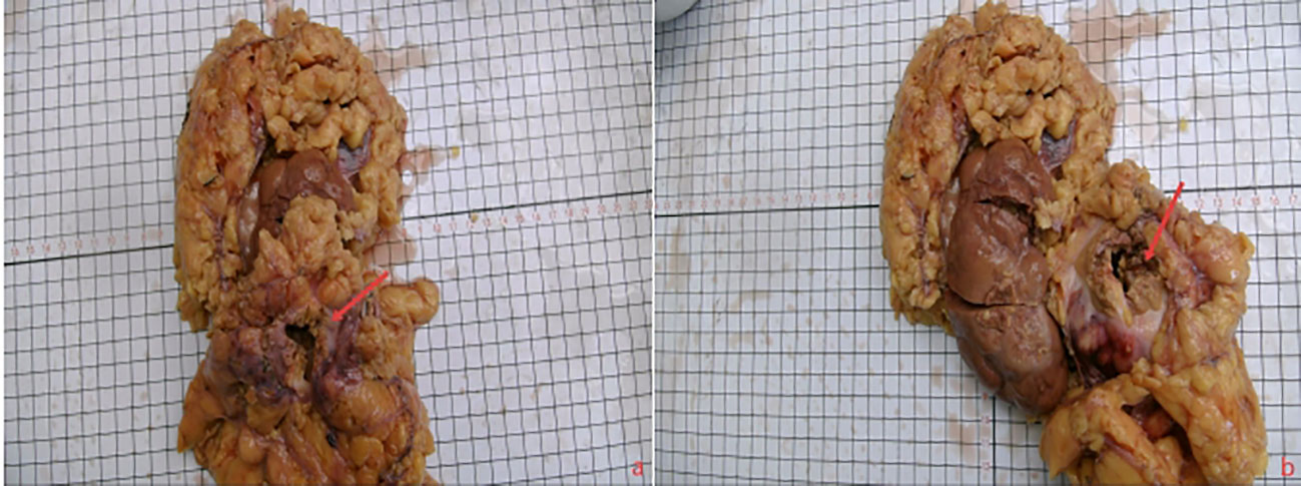
This is a general photo of the IMT of the kidney. The arrow in the picture points to the tumor location. Panels **(A, B)** are photos after the tumor was separated from the kidney.

**Figure 3:**
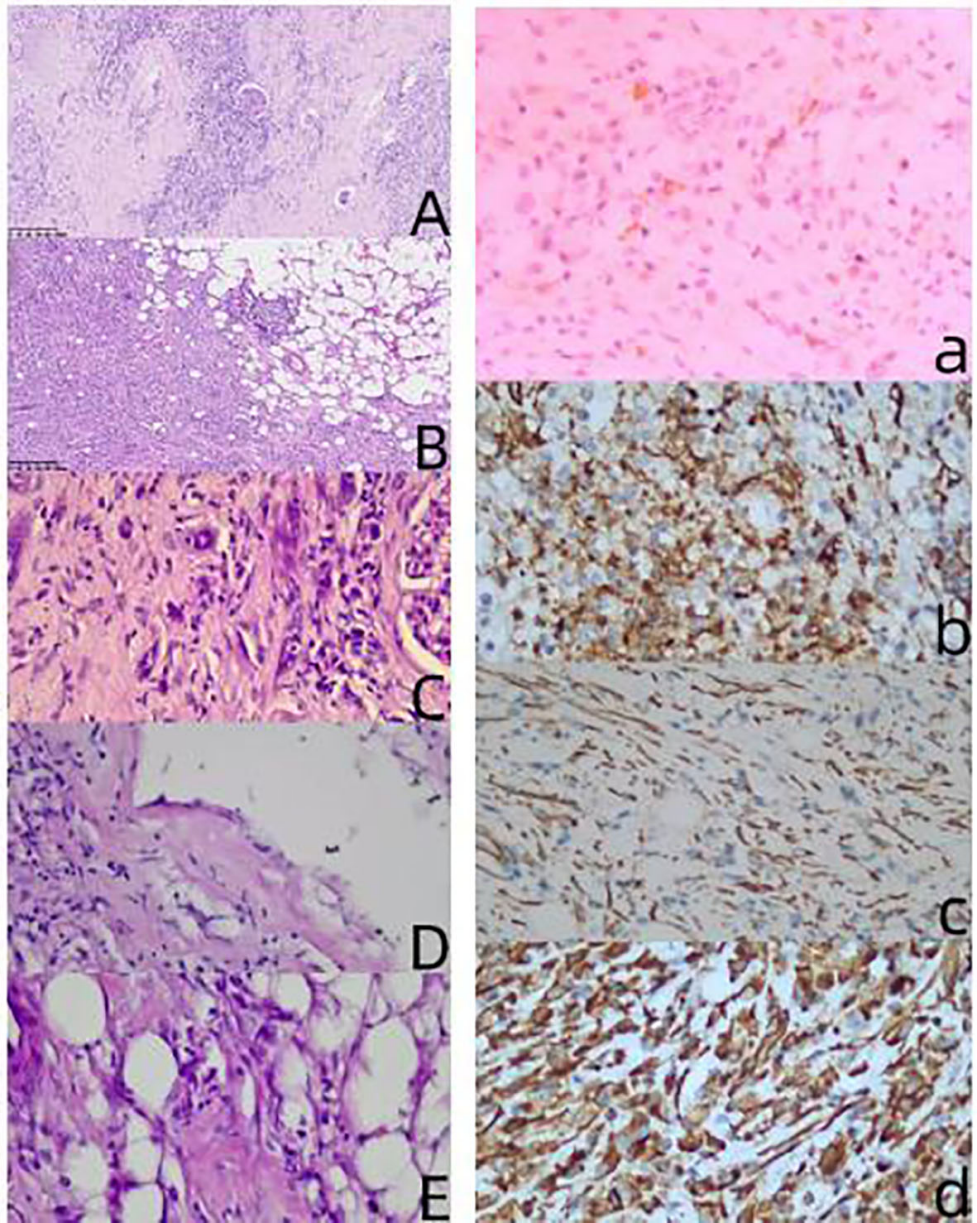
This image shows HE staining images and immunohistochemical images of IMT. Panel **(A)** is an image of the tumor involving the renal parenchyma (10×); Panel **(B)** is an image of the tumor involving the renal capsule and perirenal fat (10×); Panel **(C)** is an image of the tumor involving the renal parenchyma (40×); Panel **(D)** is an image of the tumor involving the renal capsule (40×); Panel **(E)** is an image of the tumor involving the perirenal fat (40×); Panel **(a)** is an image of the tumor immunohistochemistry *ALK* (+) (40×); Panel **(b)** is an image of the tumor immunohistochemistry CD10 (+) (40×); Panel **(c)** is an image of the tumor immunohistochemistry SMA (+) (40×); Panel **(d)** is an image of the tumor immunohistochemistry Vimentin (+) (40×).

This case involved a 63-year-old female patient who presented with intermittent abdominal pain for six months. Abdominal contrast-enhanced CT imaging (**Figure 1**) revealed a solid mass located in the anterior lip of the right kidney, exhibiting internal cystic necrosis. The lesion demonstrated indistinct margins with the right anterior renal fascia and the descending duodenum, raising a concern for malignancy. Additionally, both kidneys exhibited irregular contours, and splenic infarction was identified. Following a multidisciplinary team (MDT) discussion within the department, a high suspicion of a malignant renal neoplasm was established. Consequently, a radical right nephrectomy was performed under general anesthesia. Gross examination (**Figure 2**) revealed: (Right kidney and tumor) A single resected kidney with attached perirenal fat, measuring 16 cm × 11 cm × 8 cm. The renal fat capsule had been completely stripped. Within the renal capsule and adjacent perirenal fat, a grayish-yellow, ill-defined area measuring 4 cm × 3 cm × 2 cm was identified. On the cut section, the lesion appeared grayish-yellow, with medium consistency and poorly demarcated borders; it infiltrated the renal parenchyma but spared the renal pelvis. A segment of the ureter—7.5 cm in length and up to 0.5 cm in maximum diameter was also resected. Pathological diagnosis (**Figure 3**) indicated that the IMT in this case was a single mass on the right side of the kidney with unclear boundaries from the surrounding tissues. The lesion mainly involved the perirenal fat, the renal capsule and part of the renal parenchyma. Microscopically, the histological features were characterized by a large number of histiocytes, lymphocytes, neutrophils and plasma cells in the renal capsule and perirenal fat tissue, accompanied by the proliferation of fibroblasts and myofibroblasts, local collagenous degeneration and granuloma formation. No mitosis was observed in the lesion, and only mild tissue necrosis was observed. The unaffected renal parenchyma maintained a normal renal structure, with visible normal nephrons and collecting systems. Based on its histological features, the lesion was considered IMT, nodular fasciitis, gastrointestinal stromal tumor, inflammatory fibroblastic adenomatous polyp other spindle cell tumors and reactive proliferative diseases. Immunohistochemical staining of the pathological specimen showed Vimentin (+), CD10 (+), SMA (partially +), *ALK* (occasionally weakly +), CD68 (histiocytes +), CD3 (T cells +), CD20 (B cells +), CD38 (plasma cells +), CK broad (-), PAX-8 (-), MelanA (-), Ki-67 (index 8%). As the other diseases did not have the feature of *ALK* positivity, the patient was finally diagnosed with an inflammatory myofibroblastic tumor. After the operation, the patient was given symptomatic treatments such as fluid replacement, stomach protection and anticoagulation. The patient recovered well postoperatively and was discharged after all vital signs were stable. The patient returned for follow-up visits at 3 and 6 months after the operation, respectively, and underwent plain CT scans of the chest, lower abdomen and pelvis as well as blood biochemical tests. CT scans showed no recurrence at the primary tumor site or metastasis to other sites, and no abnormalities were found in the blood biochemical tests.

## Discussion

Inflammatory myofibroblastic tumor (IMT) is a rare mesenchymal tumor with moderate malignant potential, accounting for less than 1% of all soft tissue tumors. The first IMT was reported in 1939. Owing to its rarity and histological atypia, it was once called “inflammatory pseudotumor”, “plasma cell granuloma”, or “inflammatory fibrosarcoma”. Its characteristic is the proliferation of spindle-shaped cells of fibroblasts and myofibroblasts, accompanied by dense inflammatory infiltration mainly composed of plasma cells, lymphocytes, and eosinophils (4). Currently, it is believed that IMT may be related to trauma, surgery, calculous pyelonephritis, and autoimmune diseases (5). The most common site of IMT is the lung, and in the urinary system, it most frequently occurs in the bladder. The author believes that from the perspective of renal histocytology, the kidneys are mainly composed of epithelial-derived nephrons and have a low myofibroblast content, which is why IMT is less common in the kidneys (3).

Clinical manifestations of IMT vary depending on the location of the disease. Hematuria is the most common symptom of the urinary system. Other less common clinical manifestations include infection, abdominal and pelvic pain, and obstructive symptoms. In rare cases, patients may present with systemic symptoms such as fever and weight loss (6).The patient in this case report had no typical clinical manifestations, and a right renal mass was incidentally found during physical examination at another hospital. IMT can cause inflammation. In liver IMT, laboratory tests may show leukocytosis, neutrophilia, elevated C-reactive protein, and erythrocyte sedimentation rate (7). In renal IMT, anemia, thrombocytosis, elevated erythrocyte sedimentation rate, and polyclonal hypergammaglobulinemia are often observed. On the second day of admission to our hospital, the patient underwent a routine blood test, which showed a hemoglobin level of 111g/L and platelet count of 162×109/L. All test indicators were still within the normal range.

CT and MRI are the preferred auxiliary examinations in imaging studies (8). In special circumstances, PET-CT also plays a crucial role in the staging and comprehensive assessment of tumors. However, IMT lacks characteristic imaging features. The patient introduced in this article underwent a plain and enhanced CT scan of the lower abdomen after admission, which showed a large, approximately 3.5×3.1 cm nodular lesion at the upper pole of the right kidney with uneven density and unclear boundaries. The lesion margin showed low enhancement in the arterial phase, the center was low in density with no obvious enhancement, and the lesion continued to show enhancement in the venous and delayed phases. The perirenal fascia was slightly thickened and no filling defects were found in the right renal artery, vein, or inferior vena cava.

Owing to the lack of characteristic clinical, laboratory, and imaging manifestations of IMT, postoperative pathological examination remains the gold standard for the diagnosis of IMT. The histomorphological manifestations of IMT are diverse. The main microscopic pathological features are three types of histological manifestations: 1) myxoid type: characterized by loosely arranged round and spindle-shaped cells in an edematous/mucoid stroma with significant vascular components; 2) hypercellular type: cells are hypertrophic to ganglion-like myofibroblasts, arranged in bundles or networks; 3)low cell fibrous type, also known as the fibromatous pattern, characterized by low cell density and slender spindle-shaped cells scattered in a dense collagen matrix, with scattered inflammatory cells. Because the microscopic case characteristics of IMT are similar to those of spindle cell tumors such as nodular fasciitis, gastrointestinal stromal tumors, and inflammatory fibroblastic polyps, as well as reactive proliferative diseases, immunohistochemistry should be performed to distinguish IMT from related diseases (9).

The immunohistochemical feature is that approximately 50%-70% of IMTs have *ALK* gene rearrangement, mainly located in the short arm of chromosome 2 in the p21-p23 region. In approximately 5%-10% of *ALK*-negative IMT cases, there are ROS1 and *NTRK*3 gene rearrangements, among which the ROS1 gene is commonly found in pulmonary IMT. According to the current Chinese experts, *ALK* immunohistochemical staining is recommended because of its high expression. The postoperative pathological examination of this case showed a large number of histiocytes, lymphocytes, neutrophils, and plasma cells infiltrating the renal capsule and perirenal fat, accompanied by fibroblast and myofibroblast proliferation. Combined with the weakly positive *ALK* immunohistochemistry results, this finding is consistent with the typical pathological manifestations of IMT.

According to expert consensus on the diagnosis and treatment of inflammatory myofibroblastic tumor (IMT) in China, surgical resection is the preferred treatment for resectable IMT. However, there is no clear consensus on postoperative adjuvant therapy for resectable tumors (9). In this case, the patient was suspected of having a malignant possibility on imaging, and the patient had a history of coronary heart disease, unstable angina pectoris, and aortic valve replacement, requiring long-term anticoagulant medication. The risk of partial nephrectomy was relatively high; therefore after MDT discussion in the department, radical nephrectomy on the right side was determined to be the best surgical method.

For patients with unresectable IMT, there are no clear guidelines or expert consensus regarding the treatment plan. In this case, the author believes that genetic testing should be the first step. Approximately half of IMTs carry an anaplastic lymphoma kinase (*ALK*) gene rearrangement located in the 2p23 region of chromosome 2, which leads to abnormal *ALK* expression. Relevant literature and experimental results have shown that *ALK* rearrangement is dependent on *ALK*-mediated signaling. Therefore, tyrosine kinase inhibitors can be used to treat IMT in patients with genetically identified aggressive soft tissue tumors (10).Patients with *ALK* rearrangement often have a good prognosis. Crizotinib is the preferred treatment or unresectable IMT with *ALK* gene mutations. For IMT that is not sensitive to crizotinib, lorlatinib, a new-generation *ALK* inhibitor, has shown significant therapeutic effects (11). After the approval of crizotinib, ceritinib, and other generations of *ALK*-TKIs have been proven effective in some cases, following a similar pattern to *ALK*+NSCLC, but their efficacy should be further confirmed through cohort studies (12). In some *ALK*-negative patients, *NTRK* fusion genes have been discovered. For these patients, entrectinib has been approved for the treatment of *NTRK* gene fusion solid tumors. Although we have targeted drugs for *ALK* and *NTRK* fusion genes, the exploration of gene targets for IMT is still limited, and the high cost of genetic testing is one of the reasons why large-scale clinical studies cannot be conducted, which poses significant challenges for the treatment of patients with unresectable IMT and those who do not respond to current targeted drug therapy.

The overall prognosis of IMT is good, with 5-year event-free survival (EFS) and overall survival (OS) rates of 82.9% and 98.1%, respectively (13). Relevant studies have shown that the 5-year survival rate of completely resected IMT in the lungs is 91% (14).The recurrence rate of IMT varies from less than 2% for lung tumors to 25% for extrapulmonary lesions, depending on the location and whether the tumor can be completely resected (15).Recurrence of IMT is mainly observed in patients who have not undergone radical surgery, and the recurrence rate varies depending on the site of onset. Postoperative patients should adhere to regular follow-up. In the present case, the patient recovered smoothly after surgery and was discharged without adjuvant chemotherapy. At the 3-month and 6-month follow-up visits in the outpatient department, the patient was in good condition, and no recurrence or distant metastasis was found at the primary site. There are relatively few case reports on IMT in the kidneys. This case demonstrates that radical surgical resection is the preferred treatment for renal IMT. However, large-scale studies are needed to determine whether chemotherapy is required after surgery and targeted drug treatment for unresectable renal IMT. Therefore, many questions regarding the diagnosis and treatment of renal IMT remain unanswered. In future, more molecular-targeted drugs corresponding to IMT target genes should be explored. Any breakthrough is crucial for patients with an IMT.

## Conclusion

Renal IMT is relatively rare, and lacks specific clinical manifestations and imaging features. Pathological examination is the gold standard for diagnosis. Radical resection is recommended for early stage cases, whereas for advanced cases, targeted drugs can be attempted based on the results of genetic testing. However, its prognosis remains to be verified.

## Data Availability

The original contributions presented in the study are included in the article/supplementary material, further inquiries can be directed to the corresponding author/s.
